# Distribution of macular pigments in macular telangiectasia type 2 and correlation with optical coherence tomography characteristics and visual acuity

**DOI:** 10.1186/s12886-022-02483-y

**Published:** 2022-06-13

**Authors:** Ramyaa Srinivasan, Michel M. Teussink, Kenneth R. Sloan, Rekha Priya Kalluri Bharat, Raja Narayanan, Rajiv Raman

**Affiliations:** 1 Shri Bhagwan Mahavir Department of Vitreoretinal Services, Sankara Nethralaya, Chennai, Tamil Nadu India; 2 Heidelberg Engineering GmbH, Heidelberg, Germany; 3 Department of Ophthalmology & Visual Science, University of Alabama at Birmingham, Birmingham, AL USA; 4 LV Prasad Eye Institute, Hyderabad, India

**Keywords:** Macular telangiectasia type 2, Macular pigment; Dual-wavelength autofluorescence, OCT features, Visual acuity

## Abstract

**Background:**

To estimate macular pigment values in macular telangiectasia (MacTel) Type 2 in comparison with healthy subjects in the South Indian population across different spatial profiles and to quantify the regional differences of macular pigment optical density (MPOD) in MacTel Type 2.

**Methods:**

In this prospective cross-sectional study, healthy controls and patients diagnosed with various stages of MacTel Type 2 underwent MPOD measurement using dual-wavelength autofluorescence technique with Spectralis HRA + OCT.

**Results:**

Sixty eyes of 31 healthy subjects and 41 eyes of 22 MacTel type 2 patients were included. We found an overall decrease in MPOD values in MacTel type 2 patients (-0.109, -0.11, -0.001) in comparison with healthy subjects (0.38, 0.23, 0.06) at 1°, 2° & 6° foveal eccentricities (*P* < 0.001). In various stages of MacTel type 2, the mean MPOD was found to be higher in the peripheral region compared to the central region. We found a significantly lower mean MPOD in the central region in association with specific optical coherence tomography (OCT) parameters like inner retinal cavities (*P* = 0.035) and ellipsoid zone disruption (*P* = 0.034).

**Conclusions:**

In MacTel type 2, MPOD distribution varies in different spatial profiles with higher MPOD levels in the peripheral region compared to the central region. The macular pigment levels are associated with inner retinal cavities and ellipsoid zone disruption seen on OCT.

## Background

Macular pigments (MP) such as lutein, zeaxanthin, and mesozeaxanthin are accumulated in the central retina [1, 2]. It has been shown to enhance visual performance and suggested to protect against degenerative macular disease [3]. Changes in the concentration and spatial deposition of macular pigment have been shown in certain diseases, such as age-related macular degeneration [4]. Macular pigment absorbs blue light (range ~ 400 – 520 nm, peak 460 nm); therefore inferences regarding their molecular concentration in macular tissue can be made based on the extent with which they attenuate blue light relative to light of longer wavelengths. Thus, measuring the intensity of fundus autofluorescence (FAF) simultaneously with two wavelengths, one well absorbed and the other minimally absorbed by macular pigment. This can be quantified as macular pigment optical density (MPOD); its distribution generally high at the center of fovea and decreases at approximately 6° to 8° eccentricity [5]. Previous studies on normative data shows that MPOD measurement by dual-wavelength autofluorescence (AF) has an overall good reliability and reproducibility [6, 7]. The dual-wavelength autofluorescence technique also shows good agreement with the psychophysical techniques such as heterochromatic flicker photometry and motion photometry [8–10].

Idiopathic macular telangiectasia (MacTel) Type 2 is a macular disease characterized by a slow decrease in visual acuity, metamorphopsia, and/or difficulty in reading that mostly becomes symptomatic between the 5^th^ and 7^th^ decade [11]. A distinctive abnormal distribution of macular pigments has been described in Type 2 MacTel [12–14]. In previous studies MPOD distribution in Type 2 MacTel was calculated from different techniques that includes heterochromatic flicker photometry, motion photometry, modified confocal scanning laser ophthalmoscope, fundus autofluorescence or optical reflectance, showing a reduction of MPOD in the central macular area [11, 14–21].

Helb et al. described a marked oval-shaped irregular depletion of macular pigment in the area up to 4–7 degree surrounding the fovea adjacent to a well defined area of preserved MPOD in Type 2 MacTel. This depletion starts from the temporal zone [11]. It remains unclear, if macular pigment was preserved and just redistributed or if total MPOD even increased eccentric to its central reduction. Zeimer et al. also studied the longitudinal changes in the distribution of macular pigments and found that on a 5-year follow-up, there is central reduction and peripheral accumulation of MPOD [19]. Studies have already reported the central reduction of macular pigements in MacTel type 2. The same group studied the effect of Lutein/ Zeaxanthin supplementation on MPOD and found that on supplementation an increase in pigments was detected only in areas where it was previously present at baseline [21].

Some studies have assessed optical coherence tomography (OCT) related changes in MacTel. Zeimer et al. described three patterns of macular pigment distribution in Type 2 MacTel and correlated them with OPL-INL thickness on OCT [20]. They also correlated these patterns with disease stage and visual function [18]. Micevych et al. evaluated the A/V capillary ratio as a quantifiable metric to assess and understand early capillary change in MacTel [22]. Pauleikhoff et al. investigated the role of FAF imaging in the diagnosis of MacTel. They found the common characteristic changes are loss of macular pigment, cystoid spaces, pigment plaques, neovascular membranes and ectatic vascular changes. Both inner and outer retinal defects caused an increase in FAF [23]. Wong et al. used a multiple modality imaging to examine the ocular features in Type 2 MacTel. They observed an increase in foveal autofluorescence on FAF imaging with increase in the stages of disease category 1–3 except for category 4 as it contains both areas of increased and decreased fluorescence [24]. Ong et al. investigated the relationship between disruption in different photoreceptor layers and deep capillary plexus in MacTel. They suggested that interdigitation zone (IZ) disruption may indicate early photoreceptor dysfunction that eventually progresses to ellipsoid zone (EZ) loss [25].

However to the best of our knowledge, none of the previous studies have quantitatively estimated the regional distribution (i.e., amount of central reduction and peripheral accumulation) of MPOD and correlated with various OCT features. Chew et al. reported that morphological characteristics seen on OCT appeared to have significant impact on visual acuity loss that includes the ellipsoid zone break, presence of pigments, hyper-reflectivity and neovascular proliferation. We have used a series of qualitative OCT markers and compared it with the MPOD values in MacTel [26].

In the present study, we estimated the macular pigment values in Idiopathic macular telangiectasia in comparison with healthy subjects in South Indian population across different spatial profiles using dual-wavelength autofluorescence technique. In addition we also quantified the amount of central reduction and peripheral accumulation of MPOD and assessed the relationship of MPOD distribution with different stages of MacTel type 2, its associated OCT features and visual acuity.

## Methods

A prospective study was conducted in subjects who attended outpatient clinic of tertiary eye care hospital from June 2019 to February 2021. We included 60 eyes of 31 healthy subjects and 41 eyes of 22 patients diagnosed with various stages of Type 2 MacTel of South Indian population. Approval for the study was granted by the institutional review board of Vision Research Foundation. Study protocol followed the tenets of the Declaration of Helsinki. Written informed consent was obtained from all the subjects prior to the test.

The normal healthy subjects were included according to the following criteria: best-corrected Snellen visual acuity (BCVA) of 20/20 or better, intraocular pressure between ≥ 10 mm Hg and ≤ 21 mm Hg, refractive error up to ± 4.0 diopters (D) spherical equivalent. Exclusion criteria included presence of any ocular or systemic disease, opaque ocular media, previous trauma, intraocular surgery except for cataract surgery, use of carotenoids, vitamin or antioxidant supplementation, family history of macular degeneration, and current or past smoking. Inclusion criteria for the Type 2 MacTel subjects were as follows: a clinical diagnosis of Type 2 MacTel according to findings in the ocular fundus examination and on OCT, the absence of ocular pathology other than MacTel Type 2, spherical equivalent of ± 4.0 D. All the subjects underwent a comprehensive ocular examination that includes demographic details, ocular and systemic history, visual acuity measurement, slit-lamp examination, tonometry, pupils dilated to ≥ 6 mm in diameter (using tropicamide 0.5 mg/mL), binocular ophthalmoscopy for the posterior segment examination, followed by assessment of MPOD with the Spectralis HRA + OCT (Heidelberg Engineering GmbH, Heidelberg, Germany). As the study was conducted in hospital setting, visual acuity was measured using Snellen chart. For analysis we converted the BCVA values into logMAR units.

### MPOD measurement, estimation and spatial distribution

**Fig. 1:**
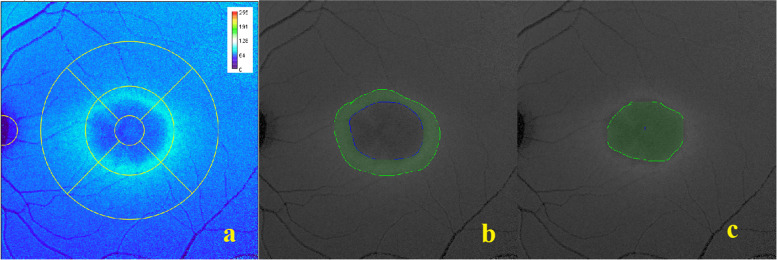
Example of the grayscale/ colourized image of **a** MacTel Type 2 subject, used to measure MPOD in two different regions (**b**—peripheral region & **c**—central region)

MPOD was measured with simultaneous dual-wavelength (excitation, 486 and 518 nm) autofluorescence in Spectralis HRA + OCT after pupil dilation. The procedure, estimation of MPOD and its spatial distribution has been described in detail elsewhere [27]. A macular pigment density map was created by Heidelberg Eye Explorer software (HEYEX, version 6.12.4.0) that shows mean MPOD and “OD sum of Volume” (i.e. MPOD pixel values in a given area are summed up), which we refer to as macular pigment optical volume (MPOV) at 1°, 2° and 6° eccentricity. The data were exported and analyzed using a customized plugin for ImageJ (National Institutes of Health, Bethesda, MD) that provides the mean MPOD values of the ETDRS grid (zone 1- central foveal; zones 2 to5—pericentral; zones 6 to 9—peripheral). Zone 1 denoted as C (central), zones 2–5 as N1, S1, T1, I1 (nasal, superior, temporal, inferior of inner ring) and zones 6–9 as N2, S2, T2, I2 (nasal, superior, temporal, inferior of outer ring) and along the 12 plots of radial sectors. It also includes a tool that enables to measure MPOD in the desired region. We used it in the images of MacTel type 2 subjects. The area was drawn manually on the grayscale or colorized image separately as the central region and the peripheral region. It appears as a horizontal ring-like structure in MacTel Type 2. The central region is seen as a dark area surrounded by a border of white color ring denoted as peripheral region (Fig. 1 a). The MPOD values were measured in density units (d.u.), and the MPOD volume corresponds to the sum of the optical density values at all points, expressed as d.u.degrees^2^.

### Clinical staging

MacTel Type 2 was clinically subcategorized into 5 stages of development according to the classification of Gass, Blodi and Yannuzzi et al. [28, 29]. Stages 1 to 3 are characterized by intraretinal changes that includes loss of transparency in the inner retina and increased visibility of telangiectasis primarily affecting the outer capillary network of the temporal fovea. Stage 1 is characterized by very few cystic changes and only mild, not clinically appreciable staining with FAF. Stage 2 by subtle loss of retinal transparency in juxtafoveal area; and stage 3 by minimal leakage into the perifoveal area with foveal cysts. Stage 3 also exhibits more extensive vasogenic process involving the fovea with prominent right-angle venules. Stage 4 is characterized by telangiectatic intraretinal vascular change within the deep and superficial capillary layers with extension toward the subinternal limiting membrane. In stage 5, development of secondary subretinal neovascularization from proliferation of intraretinal capillaries is found.

On OCT, we noted presence and absence of following abnormalities: blunting of the foveal pit, inner retinal cavities, outer retinal cavities, internal limiting membrane (ILM) draping, intraretinal hyperreflective lesions, ellipsoid zone (EZ) disruptions, foveal thinning, subretinal neovascularization (SRNV) and lamellar macular hole (LMH).

### Statistical analysis

Statistical analysis was performed using a commercially available statistical software package (SPSS for Windows, version 21.0; IBM Corp., Armonk, NY). The data were tested for normality using the Shapiro–Wilk test. The results were expressed as number and percentage for categorical data and continuous data were expressed as mean with standard deviation. Independent samples t-tests were used to check for the existence of a significant difference between the mean MPOD values in normally distributed data, and Mann–Whitney U tests were used for non-normally distributed data. Wilcoxon signed-rank test was used for pairwise comparison with the group. We considered *P* < 0.05 to be significant for these analyses.

## Results

In our study 60 eyes of 31 healthy subjects and 41 eyes of 22 MacTel Type 2 subjects were included. Remaining 2 eyes from healthy subjects and 3 eyes from MacTel Type 2 subjects were excluded due to cataract. In Control group, there were 12 males and 19 females and in MacTel group, there were 4 males and 18 females. In both the groups, there was a significant difference in males (*P* = 0.005) but not in females (*P* = 0.637). The mean age in control group was 39.10 ± 12.74 years (range, 21–65) and in MacTel group was 58.09 ± 10.19 years (range, 36–78). There was a significant difference in age between the groups (P < 0.001). The demographic characteristics of the participants in both the groups are summarized in Table 1.

**Table 1:** Demographic details of the study subjects

Parameter	Healthy controls	MacTel Type 2 subjects	*P* value
Subjects enrolled (n)	31 (60 eyes)	22 (41 eyes)	-
Gender			
Male, n (%)	12 (38.70%)	4 (18.18%)	**0.005**
Female, n (%)	19 (61.29%)	18 (81.81%)	0.637
Age (years), mean ± SD (range)	39.10 ± 12.74 (21–65)	58.09 ± 10.19 (36–78)	** < 0.001**
Race (%)	South Indian ethnicity (100%)	South Indian ethnicity (100%)	-
^a^BCVA, Snellen equivalent, median (range)	20/20 (20/20—20/20)	20/80 (20/600—20/20)	-
Refractive error (range)	-3.0 0DS to + 2.0 0DS	-4.0 0DS to + 2.5 0DS	-
Lens status, phakic/ pseudophakic (n)	57/ 3	29/ 12	-

^a^BCVA—Best corrected visual acuity

### MPOD comparison between control and MacTel type 2

The mean MPOD in Control and MacTel Type 2 groups were 0.38 ± 0.11 d.u. and -0.11 ± 0.14 d.u. at 1°foveal eccentricity; the mean MPOV was 792.25 ± 210.18 d.u.degrees^2^ and -211.63 ± 223.42 at 1° foveal eccentricity. Table 2 shows the mean MPOD and MPOV values at 1°, 2° and 6° eccentricity. It shows an overall decrease in macular pigment values in MacTel type 2 in comparison with healthy subjects and each showed a statistically significant difference between the two groups (P < 0.001).

**Table 2:** Macular pigment optical density at 1°, 2°, and 6° foveal eccentricity

Variable	Radius Eccentricity	Healthy subjects	MacTel Type 2 subjects	*P* value
Mean ^a^MPOD (d.u.)	1**°**	0.38 ± 0.11	-0.109 ± 0.14	** < 0.001**
	2**°**	0.23 ± 0.08	-0.11 ± 0.11	** < 0.001**
	6**°**	0.06 ± 0.03	-0.001 ± 0.02	** < 0.001**
Mean ^b^MPOV (d.u.d^2^)	1**°**	792.25 ± 210.18	-211.63 ± 223.42	** < 0.001**
	2**°**	2024.79 ± 670.60	-879.88 ± 795.26	** < 0.001**
	6**°**	4380.45 ± 1913.34	-4088.23 ± 3676.59	** < 0.001**

^a^MPOD—Macular pigment optical density

^b^MPOV—Macular pigment optical volume

### Regional differences in MPOD

**Fig. 2:**
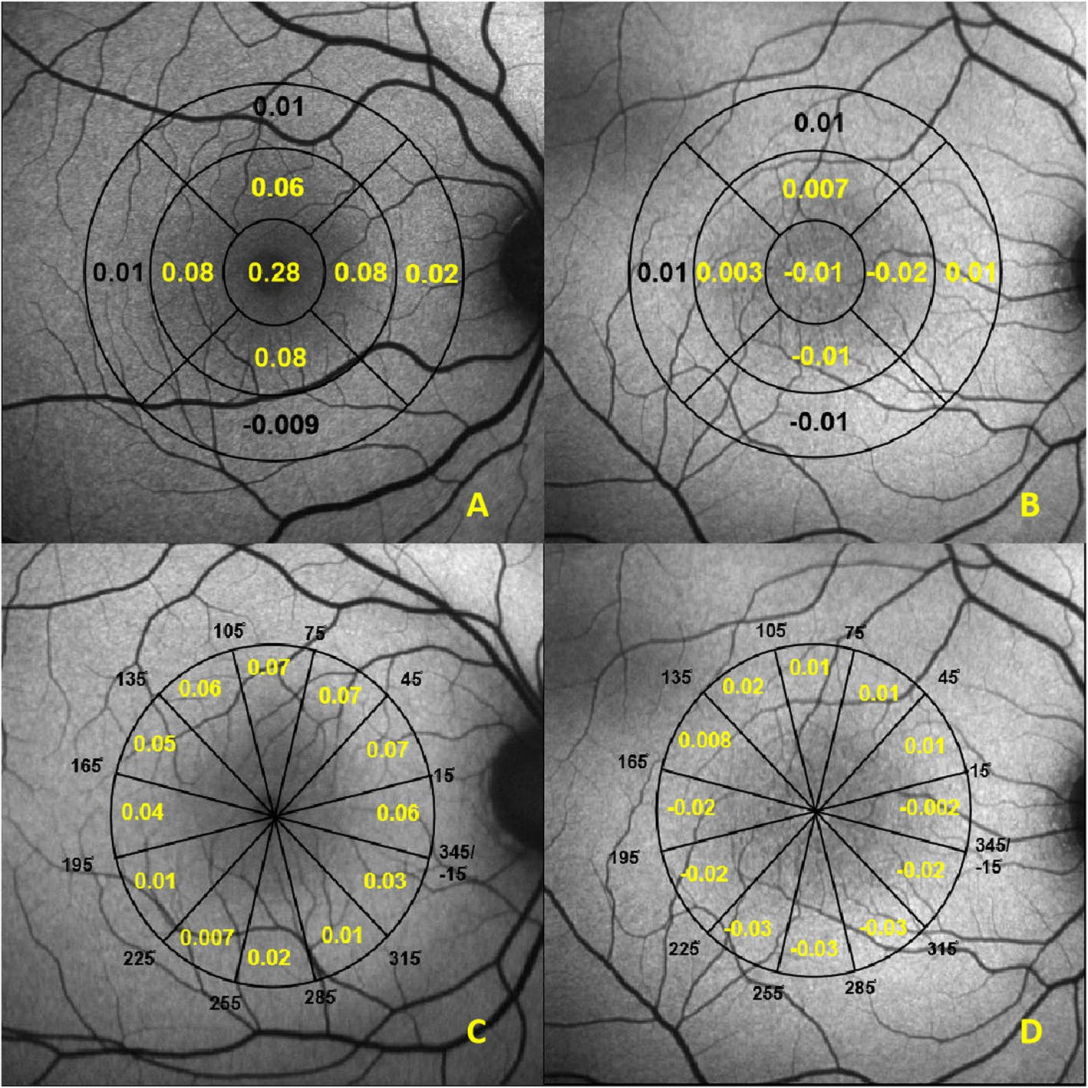
**A** and **B** Comparison of mean MPOD along the ETDRS grid between healthy controls (**A**) and MacTel type 2 subjects (**B**) **C** and **D** Comparison of mean MPOD along the 30° radial sectors between healthy controls (**C**) and MacTel type 2 subjects (**D**)(Values denoted in yellow color shows statistically significant difference of *P* < 0.05)

The distribution of mean MPOD along the ETDRS grid in both the groups are shown in Fig. 2 (A and B). In healthy controls (Fig. 2A), maximum MPOD (0.28) is found to be in the central foveal ring (C), lower in the pericentral ring and lowest in the peripheral ring. In the pericentral ring, the distribution was more or less uniform, and slightly lower in the inferior sector (I1); in the peripheral ring, the inferior sector (I2) also had the lowest mean MPOD. In the MacTel group (Fig. 2B), the lowest mean MPOD was found in the central foveal (C), inferior and nasal sectors (I1, I2, N1) and increases gradually further away from the fovea. There was a statistically significant difference between both groups in all zones of ETDRS grid except N2, I2 and T2. Figure 2 (C and D) also shows the mean MPOD along foveal eccentricity, each covering a 30° radial sector, in both the groups. In healthy controls (Fig. 2C), higher values were found in the superior compared to the inferior macula, and the lowest was found in the 225° to 255° (inferotemporal) sector. In MacTel group (Fig. 2D), positive values were found between 15° and 165° (superior half) and negative values were 165° and 345° (inferior half); the lowest was found in the -15° to 15° (nasal sector) There was a statistically significant difference of mean MPOD between both the groups in all twelve radial plots (*P* =  < 0.001).

**Fig. 3:**
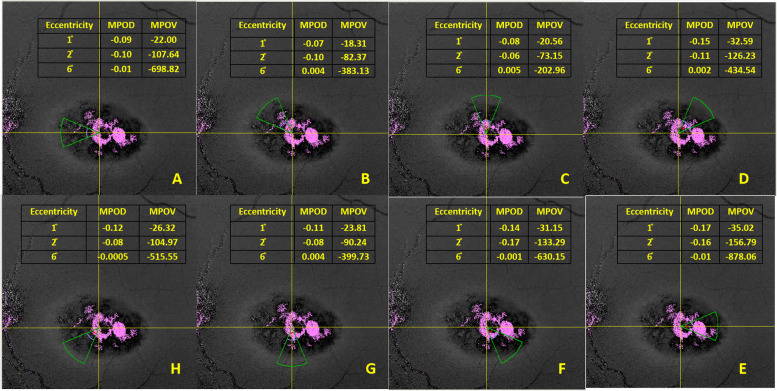
The mean MPOD and MPOV at 1°, 2° and 6° radial eccentricities in MacTel. Type 2 subjects along the clearly identifiable patterns in 8 different sectors (**A**—nasal, **B**—nasal upper, **C**—upper, **D**—temporal upper, **E**—temporal, **F**—temporal lower, **G**—lower and **H**—nasal lower)

Figure 3 represents the mean MPOD and MPOV at 1°, 2° and 6° radial eccentricities in Type 2 MacTel subjects along the clearly identifiable patterns in 8 different sectors that includes nasal, nasal upper, upper, temporal upper, temporal, temporal lower, lower and nasal lower. It shows that there was an overall decrease in macular pigments along the affected area.

The stages of MacTel Type 2 are classified based on fundus examination and OCT characteristics. In our study none of the subjects were diagnosed with stage 1 MacTel, therefore the stage 2 and 3 were combined in one group; stage 4 and 5 in another group. As exemplified in Fig. 1, in all MacTel Type 2 subjects a region was drawn manually on the grayscale/ colourized image based on the visibly well demarcated edge where MP appears to have been depleted in MacTel Type 2 within the central retina (denoted as central region) (Fig. 1 c). A surrounding ring-like structure of the adjacent region with elevated MP (denoted as peripheral region) (Fig. 1 b) was subsequently drawn, in order to determine mean MPOD in both regions. Table 3 shows the results of this analysis. In MacTel stages 2 and 3, the mean MPOD of the peripheral region was 0.04 ± 0.01 d.u. and central region was -0.02 ± 0.03 d.u. In stages 4 and 5, the mean MPOD of the peripheral region was 0.07 ± 0.12 d.u. and central region was 0.02 ± 0.25 d.u. In stages 2 and 3, as well as 4 and 5, the mean MPOD was found to be higher in the peripheral region compared to the central region (*P* = 0.001and *P* = 0.449, resp.). There were a statistically significant difference of mean MPOD in the central region (*P* = 0.020) and peripheral region (*P* = 0.011) on comparison of the stages 2 and 3 with the stages 4 and 5.

**Table 3:** Macular pigment optical density in central and peripheral regions in different stages of MacTel type 2

	^a^**MPOD**					***P* value**
	**Mean**	**SD**	**Median**	**Minimum**	**Maximum**	
Stages 2 and 3						
Peripheral region	0.04	0.01	0.04	0.02	0.08	**0.001**
Central region	-0.02	0.03	-0.02	-0.09	0.06	
Stages 4 and 5						
Peripheral region	0.07	0.12	0.04	-0.002	0.49	0.449
Central region	0.02	0.25	-0.05	-0.14	1.00	
Peripheral region						
Stages 2 and 3	0.04	0.01	0.04	0.02	0.08	**0.011**
Stages 4 and 5	0.07	0.12	0.04	-0.002	0.49	
Central region						
Stages 2 and 3	-0.02	0.03	-0.02	-0.09	0.06	**0.020**
Stages 4 and 5	0.02	0.25	-0.05	-0.14	1.00	

^a^MPOD—Macular pigment optical density

### MPOD and OCT parameters

Table 4 shows in MacTel Type 2 subjects, the association of various OCT parameters with the mean MPOD values in the drawn central region and peripheral region. In the central region, there was a significantly lower mean MPOD in subjects with inner retinal cavities (*P* = 0.035) or with EZ disruption (*P* = 0.034).

**Table 4:** OCT features and corresponding mean MPOD in peripheral and central region of MacTel type 2 subjects

^a^**OCT feature**	**Frequency** (n)	Peripheral **region** Mean ^b^MPOD (d.u.)	***P* value**	Central **region** Mean ^b^MPOD (d.u.)	***P* value**
Blunting of foveal pit					
Present	6	0.05	0.311	-0.04	0.703
Absent	35	0.06		0.009	
Inner retinal cavities					
Present	23	0.04	0.471	-0.04	**0.035**
Absent	18	0.08		0.06	
Outer retinal cavities					
Present	17	0.05	0.068	-0.03	0.206
Absent	24	0.06		0.01	
^c^ILM draping					
Present	3	0.06	0.497	-0.03	0.896
Absent	38	0.06		0.001	
Intraretinal hyperreflective lesions					
Present	23	0.05	0.582	0.02	0.572
Absent	18	0.07		-0.03	
^d^EZ disruption					
Present	35	0.06	0.164	-0.002	**0.034**
Absent	6	0.06		0.006	
Foveal thinning					
Present	15	0.06	0.349	0.03	0.891
Absent	26	0.05		-0.02	
^e^SRNV					
Present	6	0.16	0.349	-0.03	0.071
Absent	35	0.04		0.003	
^f^LMH					
Present	1	0.03	0.667	-0.06	0.595
Absent	40	0.06		0.0002	

^a^OCT—Optical coherence topography

^b^MPOD—Macular pigment optical density

^c^ILM—Internal limiting membrane

^d^EZ—Ellipsoid zone

^e^SRNV—Subretinal neovascularization

^f^LMH—Lamellar macular hole, Test done: Mann–Whitney U test

In MacTel Type 2 subjects mean MPOD (central and peripheral regions) versus BCVA was correlated. There was a positive and significant correlation of mean MPOD in the peripheral region with BCVA (R^2^ = 0.101, *P* = 0.04); in addition a negative and significant correlation of mean MPOD in the central region with BCVA was found (R^2^ = 0.108, *P* = 0.025).

## Discussion

Our study shows reduced macular pigments in MacTel Type 2 as part of their pathophysiology in comparison to healthy subjects among the South Indian population across various spatial profiles. At the three foveal eccentricities (1°, 2° & 6°), we found an overall decrease in both MPOD and MPOV in MacTel Type 2 patients in comparison with healthy subjects. Along the ETDRS grid, the distribution of mean MPOD in healthy subjects was found to be highest in the central foveal ring and decreases away from fovea; in the MacTel Type 2 patients lowest were found to be in the central foveal ring and increases away from the fovea. Along the radial sectors, in both healthy subjects and MacTel Type 2 patients, the inferior quadrants tend to have lower mean MPOD compared to the superior quadrants. In all stages of MacTel Type 2, the mean MPOD was found to be higher in the peripheral region compared to the central region. We found a significantly lower mean MPOD of the central region in subjects with inner retinal cavities and EZ disruption on OCT.

Previous studies similar to ours have described a presence of marked and well defined central depletion of macular pigments in MacTel. Issa et al., consistent with our findings, showed a lower MPOD in temporal compared to nasal quadrant [14]. The abnormal distribution of the macular pigments in MacTel Type 2 might indicate impaired trafficking or storage of Lutein and zeaxanthin in the disease process. The reason for an eccentric ring-like MP accumulation is not clearly understood. The eccentric MP in MacTel Type 2 could be due to either abnormal binding, a centrifugal displacement of central macular pigments, or a remnant of previously normal macular pigments in eccentric location.

Muller et al. evaluated macular pigment distribution pattern as a prognostic marker for disease progression in MacTel Type 2. They correlated visual acuity, microperimetry and ellipsoid zone break in OCT with classes of MPOD in Mactel Type 2 over a mean period of 5 years. At follow up, a significant decrease in visual acuity and EZ break was observed in eyes assigned to MPOD of class 2 and 3 [30]. They observed only EZ break in OCT and quantification of MPOD was not done. We quantified MPOD in different stages of MacTel type 2 along the inner and outer regions and looked into other OCT features as well. Heeren et al. reported the visual acuity and measured the size of retinal area affected by MacTel type 2. They found that neurodegeneration does not spread beyond the limits of the MacTel area. But they didn’t measure MPOD along the dimension [31]. Similar to our study, Degli Esposti et al., and Chin et al. found the abnormal paracentral distribution of macular pigments in all stages of MacTel Type 2 [16, 17]. Thus the central loss of MP doesn’t seem to be a marker of early disease. We found an increase MPOD in the peripheral region in stages 2—5 and there was a significant difference in MP densities within the peripheral and the central region in the either early/ medium or more advanced stages of MacTel.

Zeimer et al. correlated the OCT findings in MacTel Type 2 with MPOD and found that the advanced stages of MP loss were associated with thinning of outer plexiform and inner nuclear layer complex and photoreceptor layer [20]. Muller et al. also revealed a EZ loss in OCT and its progression is associated to the area of reduced MPOD [30]. Histological observations in MacTel affected eyes revealed that macular pigment loss was associated with a dysfunction or loss of Müller cells in the macular region, confirming MacTel as a possible Müller cell disease [32]. Likewise we found that EZ disruption and inner retinal cavities, were associated with lower MPOD in the inner region. Both these features are related to Müller cell damage seen in MacTel. The Müller cell cone extends to the deep capillary plexus at the border between the inner nuclear layer and the outer plexiform layer. Loss of these cells results in the inner retinal cavity lesions. The Müller cells also form tight junctions with the inner segments of the photoreceptors of which the mitochondria-rich cellular compartment appears to be visualized as the EZ band on OCT. Damage to the müller cells and EZ disruptions might lead to loss of macular pigments results in neurodegenerative changes in MacTel Type 2. There are evidences that rearrangement/ loss of macular pigments may be related to the loss or damage to the Müller cells [30, 32]. These facts probably explain the association of OCT features with MPOD levels.

Zeimer et al. studied the functional changes in the classes of MP distribution and showed a decrease in visual acuity with higher stages of MP re-distribution [18]. The same group also studied the influence of supplementation of Lutein and Zeaxanthin on MPOD [21]. They found an increase in MP after supplementation in areas where MP was present at baseline in 5–7 degree, i.e., the area we denote as the peripheral region in our study. Choi et al. studied the influence of high dose of zeaxanthin supplementation in type 2 MacTel. They reported that all 8 subjects showed an increase in MPOD in 5° to 7° eccentricity where pigments was present at the baseline. There was no enhancement in the central fovea where pigment was absent. Some patients noted subjective improvements in vision, but no objective improvements could be documented [33]. There is a conflicting evidence in the literature about whether a relationship exists between MPOD levels and dietary supplementation. We found the peripheral region macular pigment density positively correlates with visual acuity. Further studies with large sample size and longer follow-ups should perhaps investigate whether the supplementation with Lutein and Zeaxanthin could improve the MPOD in peripheral region and translate to an increased visual acuity in patients with MacTel Type 2. This study had a few limitations. The subjects in both the groups (i.e., healthy controls and MacTel Type 2) were not age and gender matched. In healthy control group, we had more number of males and subjects of lesser age group with higher MPOD values than compared to the MacTel group. That might probably led to the gender and age difference between the two groups. We did not track the dietary information or measure the serum lutein and zeaxanthin levels of the participants in relation to the MPOD levels. Microperimetry was not done to assess the preferred retinal loci and fixation stability in MacTel subjects. Future prospective studies which include concurrent multimodal imaging including adaptive optics may be required. This method relies on fluorescence to measure MPOD, some caution has to be made because other changes that can be contributing e.g. dominant fluorophore in the RPE and minor fluorophore in the neurosensory retina [34]. This could be a general failing of this technique.

## Conclusion

In summary our findings shows that, the MPOD distribution varies in different spatial profiles with higher MPOD levels in the peripheral region compared to the central region. The macular pigment levels are associated with inner retinal cavities and ellipsoid zone disruption seen on OCT.

## Data Availability

The datasets used and/or analysed during the current study are available from the corresponding author on reasonable request.

## Abbreviations

MacTel: Macular telangiectasia
MPOD: Macular pigment optical density
OCT: Optical coherence tomography
FLIO: Fluorescent lifetime imaging ophthalmoscopy
BCVA: Best-corrected visual acuity
D: Diopters
MPOV: Macular pigment optical volume
ILM: Internal limiting membrane
EZ: Ellipsoid zone
SRNV: Subretinal neovascularization
LMH: Lamellar macular hole
MP: Macular pigments
